# Exploring the ways in which COVID‐19 and lockdown has affected the lives of adult patients with anorexia nervosa and their carers

**DOI:** 10.1002/erv.2762

**Published:** 2020-07-09

**Authors:** Danielle Clark Bryan, Pamela Macdonald, Suman Ambwani, Valentina Cardi, Katie Rowlands, Daniel Willmott, Janet Treasure

**Affiliations:** ^1^ Institute of Psychiatry, Psychology and Neuroscience, Department of Psychological Medicine, Section of Eating Disorders King's College London London UK; ^2^ Department of Psychology Dickinson College Carlisle Pennsylvania USA; ^3^ Department of General Psychology University of Padova Padova Italy

**Keywords:** anorexia nervosa, carers, COVID‐19, eating disorders, qualitative research

## Abstract

**Objective:**

This qualitative study explores the ways in which the coronavirus disease 2019 (COVID‐19) pandemic and associated lockdown measures have affected the lives of adult patients with anorexia nervosa (AN) and their carers.

**Method:**

Semi‐structured interviews were conducted with patients with AN (*n* = 21) and carers (*n* = 28) from the start of UK Government imposed lockdown. Data related directly to the impact of lockdown and COVID‐19 were analysed using thematic analysis.

**Results:**

Four broad themes were identified for patients and carers separately. Patients experienced: 1. reduced access to eating disorder (ED) services; 2. disruption to routine and activities in the community; 3. heightened psychological distress and ED symptoms; 4. increased attempts at self‐management in recovery. Carer themes included: 1. concern over provision of professional support for patients; 2. increased practical demands placed on carers in lockdown; 3. managing new challenges around patient wellbeing; 4. new opportunities.

**Conclusions:**

Reduced access to ED services, loss of routine and heightened anxieties and ED symptoms resulting from COVID‐19 and lockdown measures presented challenges for patients and carers. Increased remote support by ED services enabled the continuation of treatment and self‐management resources and strategies promoted self‐efficacy in both groups.

## 1 INTRODUCTION

The coronavirus disease 2019 (COVID‐19) pandemic has had a dramatic impact on the population at large and on health services. Across the world, governments have attempted to contain the spread of the disease by placing their countries in lockdown and closing public places such as restaurants, schools and gyms. In the United Kingdom (UK) other measures have included “social distancing” (staying two metres apart from other people outside of your household) and “shielding” (protecting particularly vulnerable people at high risk of severe illness by minimizing all interaction between them and others). These measures have been highlighted as having the potential to adversely affect mental wellbeing in the short‐ and long‐term (Brooks et al., 2020; Fiorillo & Gorwood, 2020). The impact on people with current mental health problems has been identified as an urgent research priority (Holmes et al., 2020).

The impact of COVID‐19 on individuals with eating disorders (EDs) is yet to be fully determined. A preliminary survey of patients with EDs found that 38% of patients reported an increase in ED symptoms and 56.2% reported an increase in anxiety within the first 2 weeks of lockdown in Spain (Fernandez‐Aranda et al., 2020). Initial qualitative research on patients with anorexia nervosa (AN) and carers in the UK in the early phase of COVID‐19 lockdown, reported ambivalence about using video conferencing, concerns over the loss of physical monitoring and stress in managing rigid routines within the family dynamic (Fernandez‐Aranda et al., 2020). It has been suggested that limited access to certain food products as a result of food hoarding and interrupted food supply chains during the outbreak of COVID‐19 may also have been of considerable concern to individuals with EDs, particularly those already excessively limiting their food intake (Rodgers et al., 2020). Furthermore, lockdown restrictions banning gyms along with other general requirements that reduce movement, are likely to worsen feelings of restlessness in those managing compulsive exercising or using movement to reduce anxiety (Fietz, Touyz, & Hay, 2014). It has been reported that fears of virus contamination have fuelled increased health anxiety and obsessive‐compulsive disorders (OCD) in ED patients (Davis et al., 2020). Furthermore, interruptions to routine, uncertainty and social isolation may result in increased distress for individuals with EDs (Gordon & Katzman, 2020).

These early findings are of concern; they suggest that COVID‐19 and its secondary societal consequences will have an adverse impact on the health of people with EDs. In the UK, reduced face‐to‐face contact within healthcare services has been necessary during lockdown in order to protect patients and healthcare staff. However, reduced access to healthcare services may place patients at even greater risk (Holmes et al., 2020; Weissman, Bauer, & Thomas, 2020). This is of particular concern for patients with severe EDs who require substantial medical monitoring. While mental health services and therapists have rapidly turned to technology to deliver remote care (Liu et al., 2020; Weissman et al., 2020), it is also suggested that individuals with EDs may rely heavily on self‐management resources (Weissman et al., 2020) and depend more on the role of others in their household who undertake caregiving roles (Fernandez‐Aranda et al., 2020). Family carers often experience psychological distress, low self‐efficacy and high perceived burden in their caregiving experiences (Anastasiadou, Medina‐Pradas, Sepulveda, & Treasure, 2014) and it is possible that increased responsibility placed on carers as a result of lockdown, may have adverse effects on their own psychological health. This is particularly important given that carers' own emotions and behaviours can impact on patients' wellbeing (Salerno et al., 2016; Treasure et al., 2020).

Initial research suggests that COVID‐19 and lockdown may pose a particular set of challenges, unique to individuals with EDs and their carers. As such, this study aims to explore the impact of COVID‐19 and lockdown the lives of adult patients with AN and their carers.

## 2 METHODS

### 2.1 Participants and sampling

Participants were purposively selected from the TRIANGLE trial; an ongoing large‐scale multi‐centre randomized controlled trial investigating whether the addition of a novel intervention for patients and carers (ECHOMANTRA) to treatment as usual (TAU) improves outcomes for people with AN. As such, patients (*n* = 21) approached for interview were aged 16 years or older with a primary *Diagnostic and Statistical Manual of Mental Disorders* fifth edition (DSM‐5) (American Psychiatric Association, 2013) diagnosis of AN and were admitted to a specialist intensive unit at the time of consenting (inpatient or day‐patient) and had a carer (*n* = 28) willing to participate with them (Cardi et al., 2017).

The mean age of patients was 25.5 years (*SD* = 5.6), 18 of whom were female (85.7%) and the mean age for carers was 54 years (*SD* = 7.3), 23 of whom were female (82.1%). In terms of relationship to the individual with AN, 25 carers were parents (89.3%) and three were partners. Within the total sample interviewed, 44.9% (11 patients and their carers) were dyads within the study. The remainder had a carer or patient participating in TRIANGLE that was not interviewed. Overall, participants interviewed were consented from 15 different specialist units across the UK.

### 2.2 Data collection

Telephone interviews were conducted in a semi‐structured format by one interviewer (PM) and recorded using Skype. Recordings were transcribed and any identifying information was removed. The topic guides had been designed for a more extensive qualitative study for TRIANGLE with interviews beginning at the start of April 2020. However, the UK Government imposed full‐scale lockdown due to COVID‐19 on March 23, 2020. As such, the following open question was incorporated into the interview: *“What impact has the current situation had on you and your family/ loved one (COVID‐19 and lockdown)?”*. For the purpose of this study, only data that related directly to the COVID‐19 and lockdown was coded.

### 2.3 Analysis

Data were analysed using thematic analysis which is a qualitative method for identifying, analysing and reporting patterns or themes within a dataset (Braun & Clarke, 2006). Thematic analysis has proven to be a powerful tool for understanding the perspective of the patient in healthcare services (Joffe, 2012). Two researchers (PM and DCB) worked independently on the identification of themes on the entire dataset. Transcripts were read several times, and initial codes generated and incorporated into meaningful clusters before being applied to the data using qualitative analysis software: NVivo 12. An “utterance” was coded and placed in a sub‐theme, when the participant changed topic, this would constitute a new utterance and sub‐theme. In devising a final thematic framework, discussions took place between the two researchers on five occasions. Coding procedures were discussed, coding practices assessed, and agreement obtained on emerging frameworks. After each discussion, themes were either consolidated or merged into existing themes. Descriptive labels were altered to better reflect the subject matter or deleted, if deemed irrelevant to the research question.

## 3 RESULTS

Four main themes emerged from both patient and carer datasets. These are depicted in Table 1. In order to preserve anonymity, personal references have been removed from the quotations. Lockdown restrictions were set in place in the United Kingdom on March 23, 2020 and so the date that the interview took place also appears alongside the quotation.

**TABLE 1:** Themes and subthemes exploring the ways in which COVID‐19 and lockdown has affected the lives of adult patients with AN^a^ and their carers

Patients	Carers
**Reduced access to ED** ^b^ **services** Disparity in access to ED services Reliance on remote support from professionals Premature discharge from services	**Concern over provision of professional support for patients** Fears over premature discharge of patients from services Change in delivery of support for patients
2. **Disruption to routine and activities in the community** Coping with changing routine and structure Disrupted transitions into community living Reduced motivation for recovery	2. **Increased practical demands placed on carers in lockdown** Managing patient and family needs in lockdown Curtailment of normal activities and lack of routine Challenges around shielding and social distancing
3. **Heightened psychological distress and ED symptoms** Concerns over access to food and focus on exercise Increased fear and anxiety	3. **Managing new challenges around patient wellbeing** Increased displays of anxiety Reporting new food‐related triggers Spotting signs of AN relapse
4. **Increased attempts at self‐management in recovery** Experiences of increased self‐efficacy Seeking alternative practical coping strategies and resources	4. **New opportunities** Gratitude for increased time at home Noticing increased self‐efficacy Utilizing adaptive perspectives and approaches

^a^ Anorexia nervosa.

^b^ Eating disorder.

### 3.1 Patient themes

#### 3.1.1 Reduced access to ED services

Patients reported facing major challenges in accessing ED services due to COVID‐19 and lockdown‐related measures. Delivery of treatment programmes and professional support across inpatient, day‐patient and outpatient services were severely interrupted. A few patients spoke of relative successful continuation of therapeutic support delivered remotely. However, during this time there was evident disparity in access to ED services from patients' reports with varying degrees of reduction in support and physical monitoring after lockdown.

“I do get my [medical] checks done… usually it would be on a weekly basis but it's having to be on a fortnightly basis at the moment just because of the Coronavirus because I'm high risk, they don't want us sort of going out of the house…” (April 29, 2020)

Patients frequently discussed their reliance on remote support from professionals, via phone calls or video conferencing. Replacement of face‐to‐face therapy was received with varying levels of acceptability. Expressions of gratitude for the continuation of professional contact were contrasted with frustrations over inconsistent communication leaving some patients feeling abandoned by services.

“For the first week I got phone calls really regularly which was really good, I'd say they managed to keep up those phone calls since lockdown and they do really help. They definitely help but they're not the same as receiving face‐to‐face support” (April 30, 2020)

Particularly distressing reports referred to premature discharge from services. Several narratives suggested a rushed or unplanned discharge, resulting in inadequate treatment plans and fears that this may be detrimental to their recovery.

“It was so unplanned…I'd only been in four and a half weeks so obviously I hadn't been there that long and weren't anticipating discharge because there wasn't a plan in place…” (April 8, 2020)

#### 3.1.2 Disruption to routine and activities in the community

Patients described the challenges of coping with changing routine and structure. Sudden lack of routine and ability to engage in activities that provided patients with a sense of comfort, control and distraction was challenging for many who felt restless and without purpose.

“…my illness is kinda on overdrive because you can't do anything really to distract yourself from it” (April 15, 2020)

Disrupted transitions into community living after leaving inpatient treatment were also highlighted by patients as a key issue. Plans made prior to lockdown that incentivized recovery, such as socializing, living independently, returning to university or starting new jobs, had in many cases been thwarted.

“…I thought that when I come out of inpatient that I'd be going to volunteer and perhaps going back into work and I can't do that now…” (April 8, 2020)

Lack of certainty, sense of purpose and healthy distractions resulting from the COVID‐19 pandemic and lockdown resulted in reduced motivation for recovery for several patients.

“It just makes me think, oh what's the point of even trying to recover?’ because I'm not doing anything, I'm not going anywhere and it just kinda makes me a bit depressed really.” (April 13, 2020)

#### 3.1.3 Heightened psychological distress and ED symptoms

ED symptoms were noticeable in patients' concerns over access to food and focus on exercise. These predominantly featured in connection with increased feelings of anxiety related to lockdown. Food challenges centred on fears of food shortages which occurred during the initial phase of lockdown and belief that less food was required when staying at home.

“If I wasn't staying in the house, I probably would need to incorporate a snack in there somewhere as well because I would be a bit more active.” (April 29, 2020)

Patients experienced high anxiety around the topic of exercise. While some patients expressed being upset over the lack of movement due to being shielded and unable to leave the house, others struggled with controlling the urge to engage in more exercise due to the lack of other activities. In particular, patients found media attention on the potential for weight‐gain during lockdown very challenging.

“I think the media being ‘ooh you'll gain weight…’ is quite difficult because then you think ‘that's it, I'm gonna gain weight’ and I think that's one thing I've really struggled with actually…” (April 13, 2020)

Patients spoke of increased fear and anxiety around COVID‐19 that left them feeling unwell and dependent upon their ED for a sense of reassurance and control.

“My head just runs away with me so and then I worry, and then I use the ED to keep me calm and sane…” (April 24, 2020)

Furthermore, specific reports of health anxiety were described in conjunction with comments that indicated an increase in comorbid obsessive–compulsive behaviours, such as excessive handwashing. Spikes in health anxiety were also occasionally attributed to intense reporting in the media of COVID‐19.

“My OCD was hand washing and things like that, because that's got worse, that's got a lot worse since this.” (May 13, 2020)

Psychological distress was discussed by patients noticeably more than references to their physical wellbeing. Only one patient referred to extreme weight‐loss whereas descriptions of fatigue were more common and mentioned in connection to anxiety, overworking or loss of purpose.

“It's just more my moods and how I feel mentally and some days I just feel so lethargic, I cannae be bothered to get up, like I'm getting out of bed, you know?” (April 29, 2020)

#### 3.1.4 Increased attempts at self‐management in recovery

An unexpected theme emerged from the data, revealing that half of the patients interviewed found that COVID‐19 and lockdown had actually instigated increased attempts at self‐management in their ED recovery. These patients demonstrated experiences of increased self‐efficacy as a result. Patients spoke of using adaptive cognitive strategies as a means of adjusting to living in lockdown and coping with anxieties connected to COVID‐19.

“Your immune system is really weakened because of an eating disorder and for me, I don't want that anymore so I'm feeling really motivated to kind of improve my physical health as much as possible.” (May 13, 2020)

It was also revealed that some patients had proactively sought out alternative practical coping strategies and resources in lieu of their normal ED support. Patients spoke of taking on hobbies, work or volunteering opportunities to provide structure and found value in helping others during the pandemic.

“I've got like a weekly kinda list of things and I try and do some of them most days…” (April 9, 2020)

Some patients explained how lockdown had prompted them to take on more self‐management for their ED recovery through buying self‐help books and using apps to promote a proactive recovery‐oriented mindset. Participants who received the TRIANGLE intervention spoke of the timely guided self‐help resources.

“…having the opportunity to have the [TRIANGLE] forums, I think, is a really good thing, especially now with no outpatient services…” (May 12, 2020)

### 3.2 Carer themes

#### 3.2.1 Concern over provision of professional support for patient

This theme reflects carers accounts of changes to service provision brought about by COVID‐19 and lockdown. Carers expressed fears over premature discharge from services, describing a rushed transition process with inadequate treatment plans being in place for their loved ones and lack of time to prepare support, as well as concerns over the potential consequences of early discharge.

“…but I must say, that it was not a good point for her to have to leave this, of course, that was totally circumstances, that was nobody's doing, nobody's fault, but I think the last few weeks would have been really important to her to just take her right through to the last steps.” (April 8, 2020)

The majority of carers discussed their experiences of the change in delivery of support for patients. Reports included both scaled back care as well as differences in modes of care using phone calls and video conferencing. Carers expressed the challenge that these changes placed on their loved one's recovery, as well as their ability to provide support.

“They've suddenly got to weigh themselves and for those who have avoided that for a long time, and suddenly having to do it…very, very scary.” (April 7, 2020)

However, it should be noted that there were a few carers who perceived improved changes in service provision resulting in a higher standard of support than under normal circumstances, due to decreased number of inpatient admissions and increased communication.

“She's getting full support from the Unit and she knows very well that they've got beds available if the need arises, which is not a normal situation.” (April 11, 2020)

#### 3.2.2 Increased practical demands placed on carers in lockdown

The practical demands placed on carers during lockdown were diverse and considerable. Managing patient and family needs in lockdown was clearly challenging and narratives described new arrangements whereby family members now worked from home, offspring returned to the family home and generally a lack of personal space for all. Carers spoke about increased pressures and expectations on themselves, particularly as the primary carer.

“You just want some time apart and going to work does that…and you need to have that break as a carer sometimes, don't you?” (May 16, 2020)

Curtailment of normal activities and lack of routine resulting from living in lockdown was a prevalent topic among carers. There were several concerns over lack of distractions and normal lifestyle activities such as friends and social life, employment and the impact that this would have on recovery. There were concerns about the interruption to normalized eating practices, such as eating out, which carers had been hoping to encourage. There was also a recognition of their loved one's propensity towards maintaining strict routines, particularly around exercise, and the fact that this was being currently disrupted.

“Routine was very, very good for him and was a distraction from his, you know, from his obsessive circularities of thoughts connected to the eating disorder and now that has become fragmented.” (May 1, 2020)

Furthermore, some carers also raised points over the practical challenges around shielding and social distancing. These included the efforts of carers to shield vulnerable loved ones as well as issues raised by loved ones restricted from visiting their parents and families.

“What is happening is people making her feel guilty about things like her mother taking her to do some shopping.” (April 28, 2020)

#### 3.2.3 Managing new challenges around patient wellbeing

Carers showed considerable concern for their loved ones' psychological wellbeing in the context of COVID‐19. Two‐thirds of carers highlighted increased displays of anxiety in their loved ones. Increased health anxiety, general anxiety and heightened OCD were considered to be exacerbated by the threat of COVID‐19.

“[She's] got high levels of anxiety and OCD so you can imagine that, things are not easy.” (May 5, 2020)

Furthermore, carers also spoke of new food‐related triggers that have arisen due to lockdown. Challenges caused by food shortages and supermarket restrictions were described and the impact of their loved one's difficulties were felt heavily by carers who took on added responsibility for food shopping and mealtimes.

“…and it's going out to get the food and making sure that, you know, otherwise you could have a meltdown if they don't have a certain food that adds to the shopping stress.” (April 2, 2020)

Concerningly, carers also specifically described spotting signs of AN relapse in their loved one during lockdown due to the impact of early discharge, reduced support and general anxiety over COVID‐19 triggering maladaptive coping strategies.

“I mean the ED was very severe and, by the way, is getting worse under this current climate. He's really, visibly losing weight and struggling – I can see it.” (April 24, 2020)

#### 3.2.4 New opportunities

Interestingly, nearly half of carers interviewed revealed that, as a consequence of the COVID‐19 pandemic and lockdown, they had found new opportunities to provide increased support for their loved ones and utilize different adaptive perspectives and approaches in managing the challenges of COVID‐19 and lockdown. Some carers spoke of reduced pressure brought about by lockdown and expressed gratitude for increased time at home. They regarded opportunities to provide more support to their loved ones in recovery and spending time with family as valuable.

“It's almost been a benefit because I've been at home…and I'm her main support at home and normally I would be at work four days a week…but now I'm here 24/7.” (April 11, 2020)

Additionally, carers reported noticing increased self‐efficacy, both in relation to themselves and their loved ones, such as working collaboratively to come up with joint plans to manage recovery in lockdown. Some carers also expressed being proud of loved ones undertaking paid work and volunteering related to COVID‐19.

“He's now volunteering to help out with delivery of food to vulnerable people and he's absolutely loving it, absolutely loving it.” (April 6, 2020)

Finally, it was evident that despite facing such unprecedented challenges, many carers were able to use lockdown as an opportunity to utilize adaptive perspectives and approaches by adopting a resolute or constructive attitude to the situation. This allowed carers to reflect on their own needs in addition to those for whom they care, as well as offering new opportunities to experiment with different approaches to life.

“We try to be stoical about it and try to regard the present restrictions as an opportunity to redesign daily life and you know, to be more rational about all kinds of things…this is in a unusual way an opportunity to think about one's priorities about what you want to do and how you want to conduct yourself.” (May 1, 2020)

## 4 DISCUSSION

This study presents key themes that explore the ways in which COVID‐19 and lockdown affected the lives of adult patients with AN and their carers. The findings revealed that ED services across the UK had reduced provision of face‐to‐face care and moved towards delivering remote support where possible, via phone calls or video conferencing. Patients and carers shared experiences of being faced with great uncertainty and a lack of preparation for transition out of intensive treatment. The sense of being “abandoned” by services was exacerbated when promises of remote support were not delivered. However, a minority did report receiving very clear communication and support plans, delivered remotely, which provided structure for continued progression in recovery. Carers rarely discussed the direct impact on themselves as a result of premature patient discharge or reduced support. However, it was clear that carers experienced increased responsibility for looking after their loved ones who had been in hospital and had subsequently returned to the family home during lockdown.

The impact of COVID‐19 was strongly felt by patients and carers through lockdown restrictions, predominantly due to disrupted routines and ability to engage in activities, education or work. Patients' frustration at the loss of established routines and future plans frequently led to increased feelings of restlessness. Those who could leave the house experienced stress over attempts to constrain the frequency and duration of walks and exercise. Patients unable to freely leave the house, due to shielding, believed they did not need to eat as much as they normally would. This belief was said to be encouraged by media attention on the perils of reduced movement and weight gain during lockdown for the general public, an issue highlighted as potentially increasing ED risk and symptoms in previous research (Rodgers et al., 2020). While patients more frequently discussed issues around exercise than food, carers reported incidences of distress over accessing specific food that their loved ones would eat. This was mainly related to food shortages experienced in UK supermarkets at the outbreak of COVID‐19 and changes to food shopping procedures due to social distancing which increased feelings of anxiety for patients and carers. Given the central role that habitual behaviours around food and exercise play in underpinning AN (Uniacke, Walsh, Foerde, & Steinglass, 2018), the challenge of coping with the sudden and widespread disruption to daily living for patients with AN during lockdown cannot be underestimated. Furthermore, participants shared experiences of carers breaking lockdown rules to assist their loved ones with buying food or offer emotional support. This revealed the complexities of shielding vulnerable individuals with mental health issues, particularly in cases where both patient and carer were vulnerable due to illness and age, respectively.

The findings that patients reported heightened general anxiety, health anxiety and increased OCD behaviours as a direct impact of COVID‐19 was expected, given previous research demonstrating the strong link between anxiety traits, particularly OCD and AN (Levinson et al., 2019; Yilmaz et al., 2018). References to the role of media in amplifying anxiety echoes concerns expressed over repeated media consumption and health messaging around COVID‐19 for vulnerable populations (Holmes et al., 2020). While patients did not readily discuss the impact on their physical health, carers voiced more direct concern for their loved ones' physical wellbeing and highlighted signs of relapse that they themselves had noticed. Several patients revealed how lockdown had reduced their motivation to recover as well as a loss of purpose in life.

In the face of COVID‐19, increased attempts at self‐management in ED recovery were evident. Patients reported proactively turning to other sources of support, predominantly online self‐management resources and mindfulness‐based applications, in conjunction with a recovery‐oriented mindset. Having the opportunity to undertake work related to COVID‐19 was perceived to increase self‐efficacy in patients and provided a sense of purpose, supporting early findings (Fernandez‐Aranda et al., 2020). Carers also reflected on their own perspectives and approaches, with some expressing gratitude for being able to stay at home as an opportunity to offer more support and spend more time as a family. However, in sharp contrast, others confessed to feeling overwhelmed by additional caring responsibilities in addition to full‐time work, particularly with reduced support from the professional services.

## 5 CONCLUSIONS

This research provides a unique insight into the ways in which adult patients with AN and carers perceived COVID‐19 and lockdown to have affected their lives. Overall, the findings suggest that the lack of planning and resources that would normally support patients cope in transitioning from hospital care to home, combined with increased anxiety and ED concerns around health, food and exercise, resulted in an extremely challenging environment for the patients in the study. Lockdown measures also reduced patients' abilities to reintegrate into the community and motivation for recovery. The unprecedented challenges of living in lockdown were felt heavily by carers, for whom many felt increased responsibility for the wellbeing of loved ones, in addition to managing their own wellbeing and that of other family members. In cases where patients and carers fostered adaptive mindsets and self‐efficacy, they appeared to find benefit in supporting others, making the most of support offered and reaching out to alternative self‐management resources.

### 5.1 Strengths and limitations

The COVID‐19 and lockdown related question asked in this study was incorporated into a pre‐determined topic guide for the purpose of eliciting qualitative data for the TRIANGLE study (Cardi et al., 2017). The timing of the interviews provided the opportunity to discuss the impact of lockdown as it unfolded. This enabled the researchers to gain current perspectives on a rapidly evolving and urgent topic. While only one open question was directly asked on the impact of COVID‐19, prompts were used in an attempt to elicit more information, particularly if only short answers were given. However, it should be highlighted that this approach limited the ability to explore some issues raised in more depth.

A second limitation is that the results may represent patients and their carers who were more engaged and willing to share their experiences, and not the most severely unwell. However, data saturation was reached within our sample size (*N* = 49) and reflects diverse experiences of patients and carers across the UK.

### 5.2 Clinical implications

Given the projected need to anticipate long‐term adjustments to COVID‐19, it is necessary to consider the implications to services for people with EDs. Self‐management strategies for bulimia nervosa and binge eating disorder were introduced 30 years ago (Treasure et al., 1994, 1996) and these are recommended by National Institute for Health and Care Excellence (NICE, 2017). Books, smartphone applications and other online materials are available and the transfer to online individual or group guidance should be easy to implement. The adoption of this form of management to AN has been slower. For example, people were concerned that the lack of insight and ambivalence about treatment, which are characteristic of AN, might predicate against forms of collaborative care (Wilson & Zandberg, 2012). However, involving social support in AN recovery, particularly from families, has been proved to have positive effects both on carer wellbeing and reduced the need for intensive treatment (Hibbs et al., 2015; Hodsoll et al., 2017). A systematic review of these approaches found that engagement into treatment (lower drop‐out) was improved (Albano, Hodsoll, Kan, Lo Coco, & Cardi, 2019). This approach has currently been utilized in the TRIANGLE study (Cardi et al., 2017), where social support is facilitated through online support groups and self‐management resources. This study reiterates previous calls for the long‐term development and integration of online adaptive and flexible mental health resources into existing services in order to provide continued support for ED service users (Holmes et al., 2020; Weissman et al., 2020). Healthcare services will need to readjust to use more self‐management strategies for adult AN and collaborate more with sources of social and professional support, delivered remotely.

## CONFLICT OF INTEREST

The authors declare no conflicts of interest.
